# Leveraging untargeted metabolomics in combination with machine learning to uncover novel insights into bladder cancer

**DOI:** 10.1186/s40170-026-00427-4

**Published:** 2026-04-01

**Authors:** Abu Hena Mostafa Kamal, Vasanta Putluri, Tanmay Gandhi, Chandra Shekar R. Ambati, Chandra Sekhar Amara, Karthik Reddy Kami Reddy, Meredith Lauren Spradlin, Amrit Koirala, Sachin B. Jorvekar, Sandra L. Grimm, Dexue Fu, Krishna Parsawar, Felice de Jong, Chris Beecher, Subrata Sen, Seth P. Lerner, M. Minhaj Siddiqui, Yair Lotan, Livia S. Eberlin, Arun Sreekumar, Cristian Coarfa, Nagireddy Putluri

**Affiliations:** 1 Department of Molecular and Cellular Biology, Baylor College of Medicine, Houston, TX 77030 USA; 2 Advanced Technology Cores, Baylor College of Medicine, One Baylor Plaza, Houston, TX 77030 USA; 3 Department of Surgery, Baylor College of Medicine, Houston, TX USA; 4 Dan L Duncan Comprehensive Cancer Center, Baylor College of Medicine, Houston, TX USA; 5 Division of Urology, Department of Surgery, University of Maryland School of Medicine, Baltimore, MD USA; 6 Analytical and Biological Mass Spectrometry Core, University of Arizona, Tucson, AZ USA; 7 IROA Technologies, Chapel Hill, NC USA; 8 Department of Translational Molecular Pathology, The University of Texas MD Anderson Cancer Center, Houston, TX USA; 9 Scott Department of Urology, Dan L. Duncan Cancer Center, Baylor College of Medicine, Houston, TX USA; 10 Department of Urology, University of Texas Southwestern Medical Center, Dallas, TX USA; 11 Center for Precision Environmental Health, Baylor College of Medicine, Houston, TX USA

**Keywords:** Untargeted metabolomics, Orbitrap IQ-X, Bladder cancer, Machine learning

## Abstract

**Background:**

Untargeted metabolomics has emerged as a powerful approach to uncover metabolic dysregulation associated with cancer progression. When integrated with a machine learning strategy it facilitates the discovery of key metabolic pathways and predictive biomarkers with high diagnostic and prognostic value.

**Methods:**

In this study, we employed liquid chromatography coupled to high-resolution Tribrid Orbitrap mass spectrometry to perform comprehensive metabolic profiling of bladder cancer (BLCA) as well as predict invasiveness of the disease.

**Results:**

By leveraging both in-house retention time-based MS/MS spectral libraries and commercial databases, we robustly identify over 2000 metabolites. In addition, this platform allows identification of novel pathways highlighting metabolic vulnerabilities in BLCA. The application of machine learning algorithms and advanced computational modeling uncovered metabolic signatures that differentiate BLCA from adjacent normal/benign samples and distinguish muscle-invasive from non-muscle-invasive bladder cancer. Our integrative analytical pipeline addresses key challenges in metabolomics-including high dimensionality, metabolite annotation, and biological variability-through feature selection and predictive modeling. We identify candidate metabolic markers with strong potential for early detection and characterize invasiveness of the disease and identify potential therapeutic target pathways.

**Conclusions:**

This work highlights the power of combining untargeted metabolomics with machine learning to map the metabolic landscape of BLCA and to accelerate the development of precision diagnostics and future therapeutic strategies.

**Supplementary Information:**

The online version contains supplementary material available at 10.1186/s40170-026-00427-4.

## Introduction

Metabolomics has emerged as a powerful tool for elucidating metabolic alterations underlying disease processes, offering unique insights into pathophysiology and biomarker discovery. Unlike genomics or proteomics, metabolomics captures real-time dynamic snapshots of cellular activities, reflecting the complex interplay of genetic, environmental, and lifestyle factors. By profiling small-molecule metabolites in biological systems, this approach enables the identification of dysregulated pathways that contribute to disease progression and may serve as novel therapeutic targets [1].

Among metabolomics strategies, untargeted metabolomics has gained importance because it enables comprehensive detection of a wide range of metabolites without prior assumptions. This capacity is especially valuable in cancer research, where it facilitates the discovery of cancer-specific metabolic signatures and novel metabolic markers [2]. The integration of high-throughput liquid chromatography-mass spectrometry (LC-MS) has significantly enhanced untargeted metabolomics, enabling simultaneous detection of a broad spectrum of metabolites in biological matrices [3].

Despite its advantages, untargeted LC-MS metabolomics presents several challenges, including complexities in data interpretation, variability in sample preparation, and difficulties in metabolite identification. The extensive datasets generated necessitate sophisticated computational tools for robust analysis. Additionally, inconsistencies in sample processing can introduce biases, affecting reproducibility. Metabolite identification remains a significant challenge due to the structural diversity of metabolites and the limited availability of comprehensive spectral libraries. Furthermore, LC-MS-based methodologies are subject to limitations such as ion suppression effects and restricted dynamic range, which may impact the accuracy of quantification. Additionally, the structural diversity of metabolites and the limited scope of spectral libraries hinder the comprehensive identification of metabolites in biological matrices [4–6].

Bladder cancer (BLCA) is a common and clinically heterogeneous malignancy, representing a significant global health burden [7, 8]. While progress has been made in diagnosis and therapeutic approaches, outcomes remain variable, with five year survival of 71% for localized disease, 39% for regional, and 8% for distant disease [9, 10]; particularly due to a lack of molecular stratification tools. A deeper understanding of metabolic reprogramming that accompanies BLCA development and progression could inform effective diagnostic, prognostics, and therapeutic implications [11, 12].

Although previous studies have uncovered metabolic alterations in bladder cancers, many have relied on targeted metabolomic approaches or lacked the resolution and depth necessary to characterize the metabolic landscape [11–18]. There remains a critical need for comprehensive, untargeted studies that can capture the dynamic and diverse metabolic aspects of BLCA metabolism and progression.

In this study, we applied an advanced LC coupled to high-resolution MS to identify global metabolomics alterations in BLCA and to characterize the aggressiveness of the disease. By combining cutting-edge analytical instrumentation with robust machine learning (ML) approaches, we aim to identify metabolic signatures that enhance our understanding of BLCA biology and support the advancement of precision medicine, improving diagnosis, risk stratification, and clinical outcomes of BLCA.

## Materials and methods

### Clinical samples

For this study, human bladder cancer (BLCA) and adjacent normal/benign tissues were obtained in a de-identified manner from the Cooperative Human Tissue Network (CHTN), the University of Maryland Baltimore (UMB), and the University of Texas Southwestern Medical Center (UTSW). All samples were collected in de-identified manner under approved IRB protocols and stored at −140 °C until further analysis. The clinical information used for the study was provided in Supplementary Table 1.

### Sample preparation and metabolite extraction

Approximately 10 mg of tissue was homogenized in 300 µL of a 1:1 (v/v) methanol-water solution on ice (4°C). The lysate was transferred to a 2 mL Eppendorf tube, and 900 µL of methanol-acetonitrile (1:1, v/v) was added (3-fold volume relative to the lysate). The mixture was vortexed for 5 minutes, then incubated at −20 °C for 20 minutes to precipitate proteins and other insoluble components. After incubation, the samples were centrifuged at 15,000 rpm (4 °C, 10 min). The supernatant was transferred into 2 mL tubes and dried under a speed vacuum (GeneVac, SP Scientific). The dried samples were reconstituted in 100 µL of methanol-water (1:1, v/v), followed by vortexing (5 min) and water bath sonication (5 min) to ensure complete solubilization. The samples were then centrifuged again (15,000 rpm, 4 °C, 5 min) to remove any particulates. For quality control (QC), 10 µL aliquots from each sample were pooled and used for this study.

### Liquid chromatography-mass spectrometry

The study was conducted utilizing the Thermo Scientific Vanquish UHPLC system (Thermo Fisher Scientific), which was equipped with a Vanquish Horizon Binary Pump H, a Vanquish Column Compartment H, and a Vanquish Split sampler HT. Metabolites were separated using Hydrophilic Interaction Liquid Chromatography (HILIC) with an ACQUITY UPLC BEH Amide Column (130 Å, 1.7 µm, 2.1 mm X 150 mm) from Waters Corporation, as well as Reverse Phase (RP) chromatography utilizing an ACQUITY UPLC HSS T3 Column (100 Å, 1.8 µm, 2.1 mm X 150 mm), both in electro spray ionization (ESI) positive and negative modes [19]. The Thermo Scientific Orbitrap IQ-X Tribrid mass spectrometer from Thermo Fisher Scientific is operated using IQ-X Tune instrumental control software. Real-time data is collected through Thermo Xcalibur Software, utilizing ultra-high purity nitrogen and helium gas. This instrument offers high-resolution accurate mass (HRAM) analysis, coupled with quadrupole isolation and fragmentation of precursor ions achieved through high-energy collision-energy dissociation (HCD) through Orbitrap. This fragmentation process generates spectra containing fragment ions in both ESI positive and negative modes for comprehensive detection. Data acquisition occurs in two modes: full scan MS mode and MS^2^ mode via data-dependent acquisition (DDA).

### Quality controls

The injection sequence in LC-MS is carefully planned to ensure system readiness, assess quality, monitor control parameters, and normalize analytes to the reference matrix. A detailed sequence table is prepared, outlining sample details, acquisition method, and sample locations for the LC autosampler. Before initiating the analysis of biological samples, a series of blank samples are run to condition the system and remove any residual matrix from the column and LC system, minimizing the risk of analyte carryover. Following this steps, pool quality control (QC) samples are injected to optimize column performance in preparation for analyzing biological samples. Pool QC samples are strategically injected at the beginning and end of the sample batch and at intervals of the samples throughout the acquisition process. This ensures consistent monitoring of system performance and quality across the entire batch to prevent any potential bias caused by temporal drift in instrument performance. Biological samples are randomized before analysis, ensuring accurate comparison of metabolite abundances during subsequent data analysis.

### Generation of the In-house retention time-based MS/MS spectral library

An in-house spectral library was built using reference standards of 630 metabolite compounds from IROA Technologies. The compounds were prepared according to the manufacturer’s protocol and analyzed using a Thermo IQ-X Orbitrap Tribrid mass spectrometer coupled to Vanquish Horizon LC. Data acquisition was performed in both ESI positive and negative modes using four LC methods: HILIC-Positive, HILIC-Negative, RP-Positive, and RP-Negative. The LC system was coupled to the mass spectrometer with an optimized gradient and mobile phase composition for each method. Mass spectra were acquired in DDA mode with high-resolution full MS and MS/MS scans. The acquired spectra were processed using MLS Discovery software.

After review, based on metabolite peaks detected using MLS Discovery software, 518 unique metabolites out of 630 were qualified for inclusion in the MS/MS spectral library. Compound identification was confirmed, and an in-house curated spectral database was created as NIST file (.msp), then converted to Thermo Compound Discoverer (CD) format (.db) for use as a plug-in the library for database search. Identified spectra were annotated with retention time, accurate mass, and MS/MS fragmentation patterns (Supplementary Figure 1). Additionally, adducts were also considered when generating the spectral database.

### Metabolite identification and statistical analysis

Compound Discoverer (CD), version 3.3, serves as an effective tool for qualitative and semi-quantitative analysis in untargeted metabolomics. It leverages accurate mass data, isotope pattern matching, fragment matching, and mass spectral library searches to identify the structural composition of small molecules, including chromatogram alignment. CD boasts a user-friendly and customizable node-based processing workflow tailored to handle Xcalibur RAW files. This workflow seamlessly processes raw data into single result files while facilitating statistical analysis, thus streamlining the metabolomics data processing pipeline. Processing raw files through CD involves several sequential steps. Initially, spectra were extracted from the raw data, followed by retention time (RT) alignment with a tolerance of 0.2 minutes, peak detection tolerance of 1.5 minutes and a mass tolerance of 5 ppm. In a subsequent stage, missing values were imputed using CD automatic workflow with a Fill Gaps node for further data processing. For metabolite identification, CD utilizes m/z information from multiple MS/MS Spectral databases; therefore, naming of the metabolites may vary between databases. This applies to the in-house reference library as well as public spectral libraries such as mzCloud and the NIST Mass Spectral Library. These processes utilized QC samples and compound identification to ensure analytical consistency and accuracy. The identified compounds were annotated utilizing various online and offline databases, including an in-house RT-based library, online mzCloud, NIST 2020, and mass list from Human Metabolome Database (HMDB). To enhance identification accuracy, the mzLogic algorithm utilized all available fragmentation scans (full MS^n^ depth) for an compound [20], scoring potential matches. In cases where mzCloud search results did not yield any matches for an unknown compound, the mzLogic algorithm provided ranking scores based on identification. Adducts were taken into account during the identification process. Additionally, the algorithm provided ranking scores for different database search results when an unknown compound had available data-dependent MS^2^ scans and similarity results from spectral libraries search.

Metabolites were matched against the in-house RT-based MS/MS spectral library, with higher priority given to multiple matches over single matches. Metabolites were categorized as “A” when they matched not only the “retention time and MS/MS spectra in our in-house IROA-based reference library” but were also spectral matched with either the “mzCloud MS/MS spectral libraries” or “NIST MS/MS” spectral libraries. Features were categorized as “B” when they matched either the “mzCloud MS/MS spectral libraries” or “NIST MS/MS” spectral libraries. The features were categorized as a “C” when they matched only the accurate mass from a curated mass list (HMDB database). The names of metabolites are not assigned exclusively from our in-house RT-based spectral library. During annotation, CD prioritizes matches from integrated databases such as mzCloud and ChemSpider when assigning metabolite names. As a result, even when a metabolite is confirmed using our in-house retention time and MS/MS reference library, the reported name may reflect the preferred match selected by the software from mzCloud or ChemSpider rather than the alternative name (e. g., isomeric name/synonym name/common name/IUPAC name) used in our in-house library for category “A”. Therefore, the metabolite names in our in-house RT-based spectral library may differ from those reported by mzCloud and ChemSpider, and the final reported name typically follows the mzCloud or ChemSpider database entry.

In cases where metabolites matched with the same score, the lowest coefficient of variation (CV) of pool QC was used to determine the best match. High mass accuracy was maintained as the standard. After filtering the data, the drug metabolites were further filtered using the DrugBank Database 2023 [21] (DRUGBANK; https://go.drugbank.com/) to exclude the known drugs, since the current study focused on solely metabolomics.

The peak areas from four different methods including RP-Positive, RP-Negative, HILIC-Positive, and HILIC-Negative, were normalized using a method-wise median interquartile range (IQR) normalization method as previously described [19]. Altered metabolites were identified using a t-test followed by the Benjamini-Hochberg False Discovery Rate (FDR) test [22], considering an FDR of less than 0.25 as indicative of differentially altered metabolites [12, 16, 19, 23–27]. For the analysis, BLCA pathological stages Ta and T1 were classified as non–muscle invasive bladder cancer (NMIBC), whereas stage T2 and higher (>T2) were classified as muscle invasive bladder cancer (MIBC). After filtering the data, we used an R package-based comprehensive pipeline (https://github.com/CoarfaBCM/runModac) to help normalize the data and perform the required statistical analysis, including ensuring data quality.

### Pathway enrichment analysis

The metabolites with significant alterations between adjacent normal/benign and BLCA as well as MIBC and NMIBC patient tissues were selected for the metabolic pathway analysis. Differentially expressed metabolites were mapped to associated genes using the HMDB database [12, 16, 28]. These identified genes were then used for pathway enrichment analysis through over-representation analysis [29–31] (ORA, refer to supplementary table 2–9) using the KEGG [32], Reactome [33], Hallmark [34], and GO [35] databases.

### Machine learning models

For supervised machine learning analyses, normalized untargeted metabolomic profiles were used. Machine learning (ML) models were derived using k-nearest neighbor (KNN), Random Forest (RF), and Support Vector Machines (SVM) [31, 36, 37]. A cross-validation approach was used, with data split into 80% training for model building, then 20% for model testing; the R package caret was used to implement the cross-validation approach [38]. Performance of classification was quantified using the Area Under the Receiver Operating Characteristic curve (AUROC). The training/testing split was conducted over 100 cross-validation iterations, and the median value and distribution of AUROC were collected. The importance of individual features (metabolites) was determined using the Interpretable Machine Learning R package [38, 39], and features were further filtered for those informative in least 70% of iterations. For the best performing model for each machine learning problem, the top 20 informative features were reported, together with their direction of association with the outcome, positive or negative. Lastly, models were derived using top 2–20, 25, 30, 40, 50, 75, and 100 most informative features to determine a minimum complement of features sufficient to classify the outcomes.

## Results

**Fig. 1:**
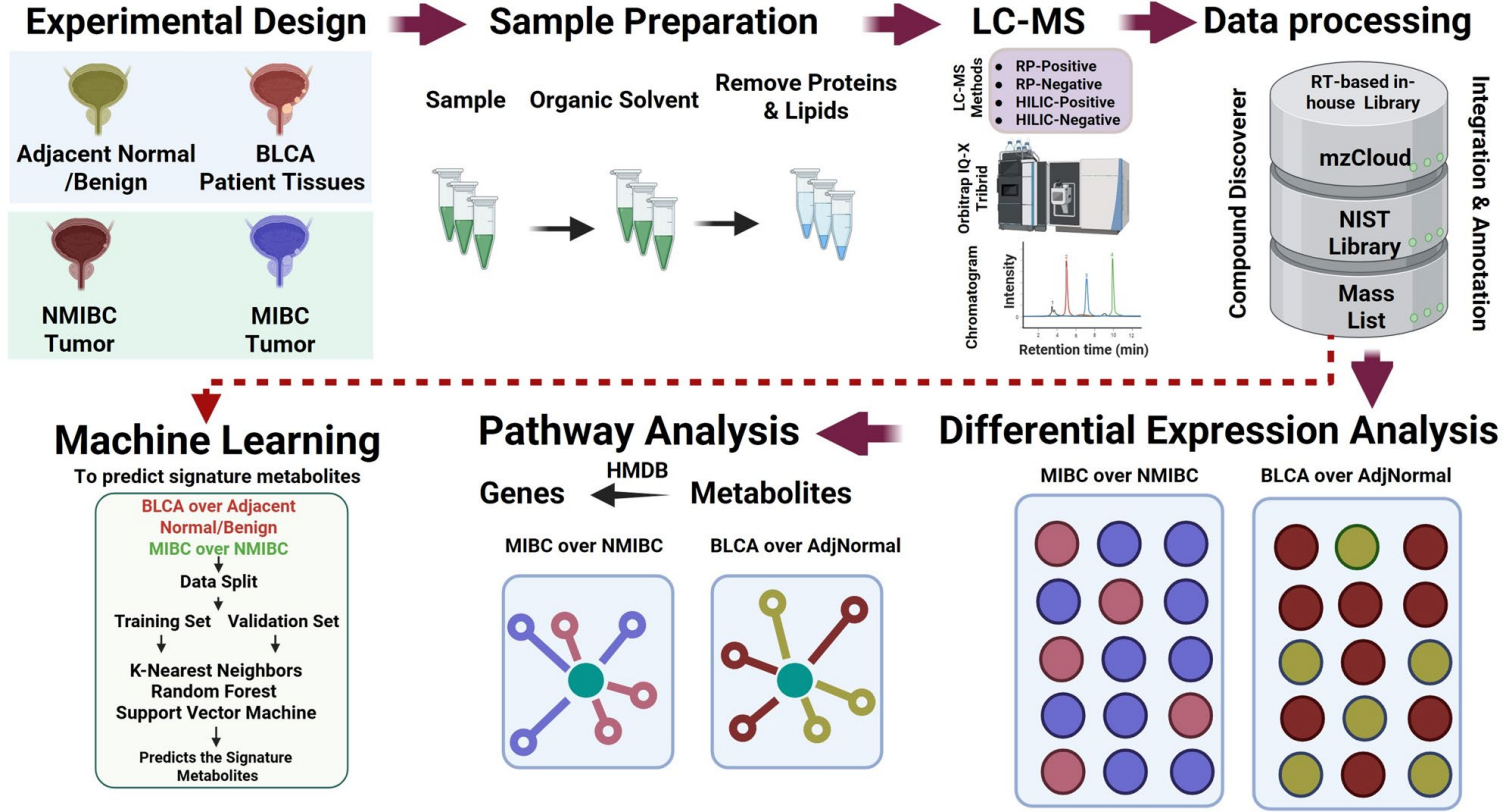
Schematic diagram represents the entire workflow used for the study

We developed a robust untargeted metabolic profiling platform using high-throughput liquid chromatography-mass spectrometry (LC-MS) with the Thermo Scientific IQ-X Orbitrap. This platform was meticulously designed to capture a comprehensive metabolic fingerprint of biological samples by incorporating four distinct separation and detection methods: reverse-phase (RP) in both positive and negative ion modes (RP-positive, RP-negative) and hydrophilic interaction liquid chromatography (HILIC) in both positive and negative ion modes (HILIC-positive, HILIC-negative). We developed an in-house retention time (RT)-based spectral library using standard compounds to characterize metabolites via LC-MS method. This in-house spectral library was integrated with external databases such as mzCloud and NIST to enhance metabolite identification. This multi-modal approach enabled a broad coverage of metabolites, encompassing both polar and non-polar compounds, thereby enhancing the detection sensitivity and overall analytical depth (Fig. 1).

Following data acquisition, the mass spectra were processed using the Compound Discoverer (version: 3.3) software, which facilitated the identification of metabolites by searching against several mass spectral libraries. These libraries included an online mzCloud, NIST 2020, an in-house RT-based library, and a mass list from Human Metabolome Database (HMDB). The comprehensive use of these libraries ensured highly accurate measurement in metabolite identification, allowing for extensive coverage of the metabolome. A rigorous data filtering and quality assurance process was implemented to ensure the accuracy and reliability of the identified metabolites. The data processing workflow included filtering and MS/MS-based identification (Category A-B), emphasizing library matches and mass accuracy. Metabolites that did not identified a definitive match in the spectral libraries were further subjected to metabolites annotation from Category C (Mass List). When multiple library matches had the same score, the metabolite with the lowest CV among the QC samples was selected as the best match. The above filtering criteria allowed for the inclusion of metabolites in all levels that, although not fully characterized by spectral libraries, were likely to be true positives due to their consistent detection across replicates. As shown in Supplementary Figure 2, the QC samples shows robust reproducibility, with correlation coefficients > 75% for peak areas (Supplementary Figure 2A) and their corresponding histogram (Supplementary Figure 2C). After normalization, the QC data show even higher correlations (>94%) with consistent signal distribution and stability across injections (Supplementary Figure 2B and 2D).

### Assessment of the untargeted metabolomics platform

LC-MS analysis was performed to profile the global metabolome, including adjacent normal/benign tissues, and BLCA patient tissues (Supplementary Table 1). We employed a liquid-liquid extraction method using acetonitrile and methanol to extract metabolites from tissue samples of both normal and BLCA patients. This procedure enabled the extraction of both polar and non-polar metabolites. The extracted samples were then analyzed using high-throughput LC-MS. Data analysis was performed using CD software (Thermo Fisher Scientific), utilizing multiple spectral libraries. Additionally, data acquisition was carried out using four developed LC-MS methods, and the mass spectra were searched against various spectral libraries, including in-house library and mass lists. Subsequently, the data were filtered using established criteria including different categories [40–42], and statistical analysis was conducted to identify the metabolites that were altered between the normal and BLCA patient tissue samples (Fig. 1 and Supplementary Figure 1). In each of the four analytical methods, we ran eight pooled quality control (QC) samples, distributed across the sample acquisition. Subsequent analysis revealed excellent correlations among the QC samples across all methods, as demonstrated by correlation plots (Supplementary Figure 2). These strong correlations indicate the high reproducibility and consistency of our metabolomic analyses, ensuring the reliability of the data obtained for the subsequent comparative studies between benign and cancerous tissue samples. After filtering the metabolites, we identified 2590 metabolites/compounds across all analytical methods (Supplementary Figure 3 and 4).

### Altered metabolites and their associated pathways

**Fig. 2:**
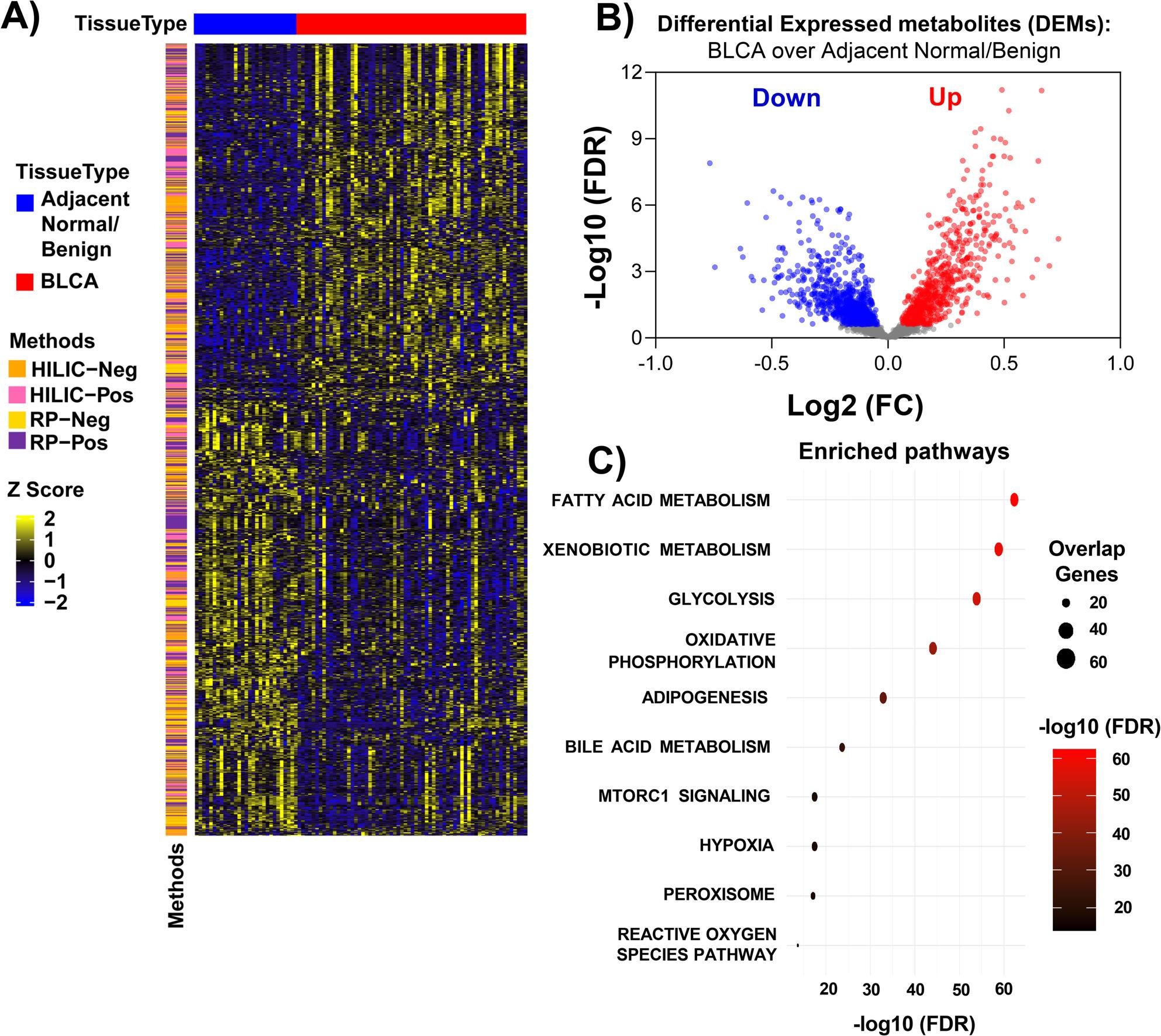
Identifying altered metabolites and key pathways in bladder cancer (BLCA) compared to adjacent normal/benign. **A**) Heatmap showing differentially expressed metabolites (DEM) in BLCA (n = 65) compared to adjacent normal/benign (n = 29) tissues (FDR <0.25). The color scale (z-score) represents relative abundance of metabolites. **B**) Volcano plot illustrating the significance and fold changes of metabolites in BLCA (n = 65) compared to adjacent normal/benign (n = 29) tissue samples. **C**) Dot plot showing the top 10 significantly enriched hallmark pathways (refer to supplementary table 2) obtained using DEMs between BLCA over adjacent normal/benign. DEMs (FDR < 0.25) were mapped to genes and used for pathway analysis. The number of genes from pathways and their significance is represented

When comparing between adjacent normal/benign (*n* = 29) and BLCA (*n* = 65) samples, a total of 1475 altered metabolites (FDR < 0.25) were identified across all analytical methods. These metabolites were detected across 4 methods and categorized as “A-C” based on the nature of the identifications (Fig. 2A and Supplementary Data 1). Out of 1475 metabolites, 812 metabolites were decreased and 663 were increased in BLCA tumor samples compared to adjacent benign/normal samples (Fig. 2B).

Next, we mapped the differentially expressed metabolites (DEMs; 1475) to genes using HMDB, then we performed over-representation analysis against the pathway collections Hallmark, KEGG, Reactome, and Gene Ontology (GO) to understand the potential functional impact of DEMs in BLCA. In Hallmark pathways, fatty acid and xenobiotic metabolism showed the most significant enrichment, indicating major lipid metabolism alterations in BLCA. Other notable pathways include glycolysis and oxidative phosphorylation, reflecting significant metabolic reprogramming. Adipogenesis and bile acid metabolism were also enriched (Fig. 2C and Supplementary Table 2). A top enriched KEGG pathway (Supplementary Figure 5A and Supplementary Table 3) was glycolysis and gluconeogenesis, consistent with the Warburg effect; cytochrome P450-mediated xenobiotic metabolism and glycerophospholipid pathways were also prominently enriched. Analysis of Reactome pathways (Supplementary Figure 5B and Supplementary Table 4) highlighted significant changes in lipid metabolism, biological oxidations, and amino acid metabolism. Gene Ontology analysis (Supplementary Figure 5C and Supplementary Table 5) showed strong enrichment for organic acid metabolism and other critical processes like oxidation-reduction and lipid metabolism. These results emphasize the extensive metabolic alterations in BLCA, particularly in carbohydrate, lipid, and amino acid metabolism pathways. The identification of these enriched pathways provides valuable insights into the metabolic vulnerabilities of BLCA, which could be exploited for the development of targeted therapies.

**Fig. 3:**
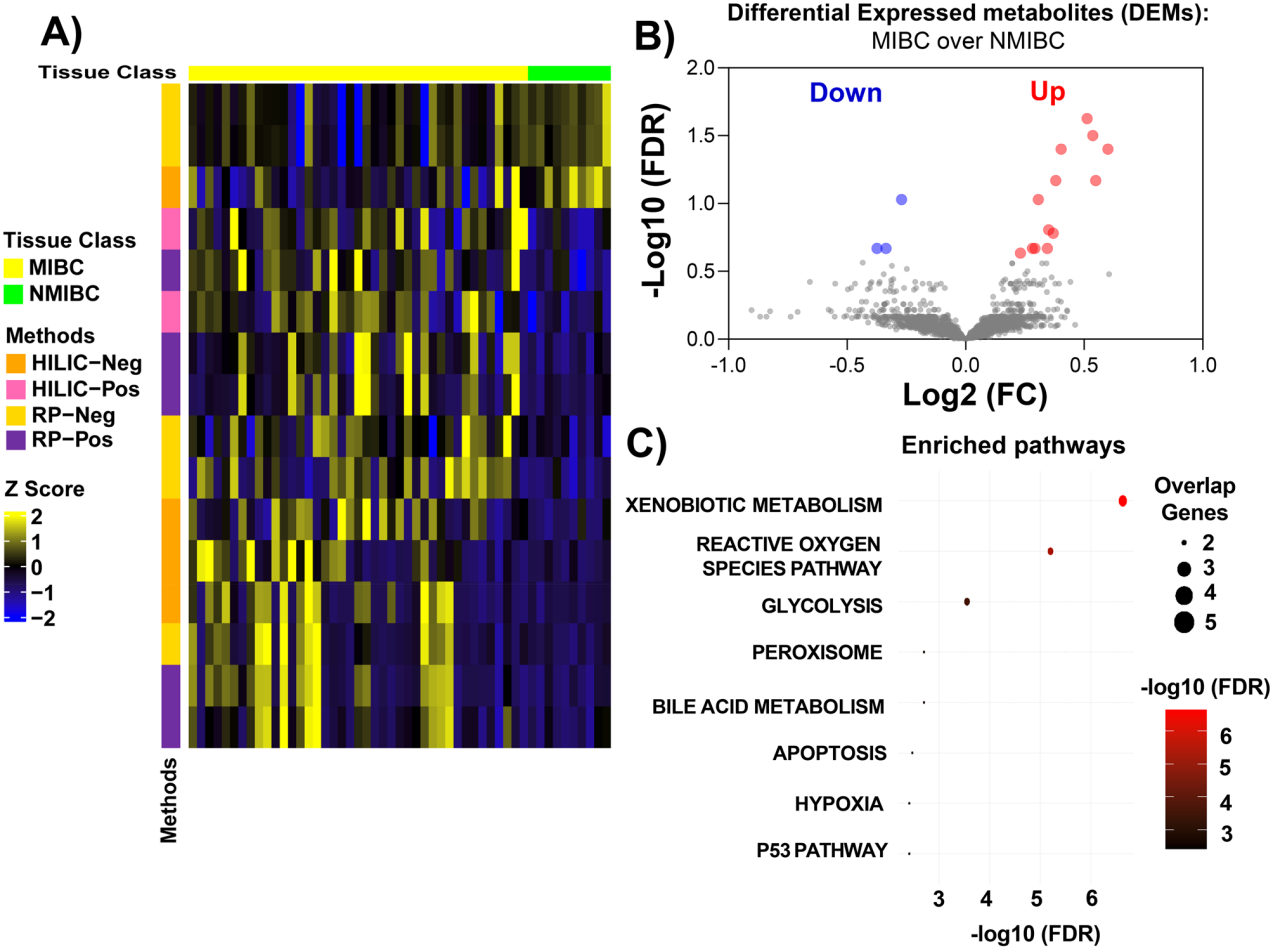
Metabolic and pathway alterations in MIBC compared to NMIBC. **A**) Heatmap showing differentially expressed, metabolites (DEM) in MIBC (n = 41) compared to NMIBC (n = 10) patients (FDR <0.25). The color scale (z-score) indicates relative metabolite abundance: upregulated metabolites are shown in yellow, and downregulated metabolites are shown in blue. **B**) Volcano plot illustrating the significance and fold changes (Log2) of metabolites in MIBC (n = 41) and NMIBC (n = 10). **C**) Dot plot showing the top 10 significantly enriched hallmark pathways (refer to supplementary table 6) obtained from DEMs between MIBC (n = 41) and NMIBC (n = 10). DEMs were mapped to genes and used for pathway analysis

In this study, BLCA pathological stage Ta-T1 tumors were considered as NMIBC, and T2–T4 tumors were considered as MIBC for the analysis. When comparing MIBC (*n* = 41) over NMIBC (*n* = 10) samples, a total of 16 altered metabolites (FDR < 0.25) were identified across all analytical methods. In the RP-Positive method, 5 altered metabolites were identified, whereas 5 in RP-Negative method, 2 in HILIC-Positive method, and 4 in HILIC-Negative method (Fig. 3A). Out of 16 metabolites, 3 metabolites were decreased whereas 13 metabolites were increased in MIBC samples compared to NMIBC samples (Fig. 3B). Next, we mapped the differential metabolites (DEMs; 16) to genes using HMDB, then we performed over-representation analysis using the Hallmark, KEGG, Reactome, and GO pathway compendia to understand the key pathways that are impacted in MIBC. Among the Hallmark pathways (Fig. 3C and Supplementary Table 6), xenobiotic metabolism exhibited significant enrichment. Other prominently enriched pathways include reactive oxygen species (ROS) signaling, glycolysis, and bile acid metabolism, highlighting key aspects of metabolic reprogramming. Additionally, apoptosis and hypoxia were also enriched, indicating broader alterations in lipid metabolism (Fig. 3C). KEGG pathway analysis (Supplementary Figure 6A and Supplementary Table 7) revealed enrichment of glutathione metabolism as well as other key pathways including cytochrome P450-mediated xenobiotic metabolism. Cytochrome P450 enzymes play a crucial role in xenobiotic metabolism by detoxifying carcinogens in BLCA as shown in our earlier publication [11]. However, their altered expression in tumors can lead to impaired detoxification, contributing to increased toxicity or drug resistance. Dysregulated P450 activity also affects hormone metabolism, influencing cancer progression and therapeutic outcomes [43–45]. Reactome pathway analysis (Supplementary Figure 6B and Supplementary Table 8) highlighted significant enrichment of glutathione conjugation. GO analysis (Supplementary Figure 6C and Supplementary Table 9) revealed strong enrichment in xenobiotic metabolism and glutathione metabolism. The findings reveal significant metabolic changes in MIBC, particularly in pathways related to xenobiotic metabolism, lipid metabolism, glycan biosynthesis. The enrichment of glutathione metabolism across both KEGG and Reactome analyses emphasizes its crucial role in detoxification and maintaining redox homeostasis. These altered pathways provide a deeper understanding of the metabolic vulnerabilities in BLCA, offering potential targets for therapeutic intervention.

### Detection of biomarker candidates for BLCA and MIBC using machine learning

**Fig. 4:**
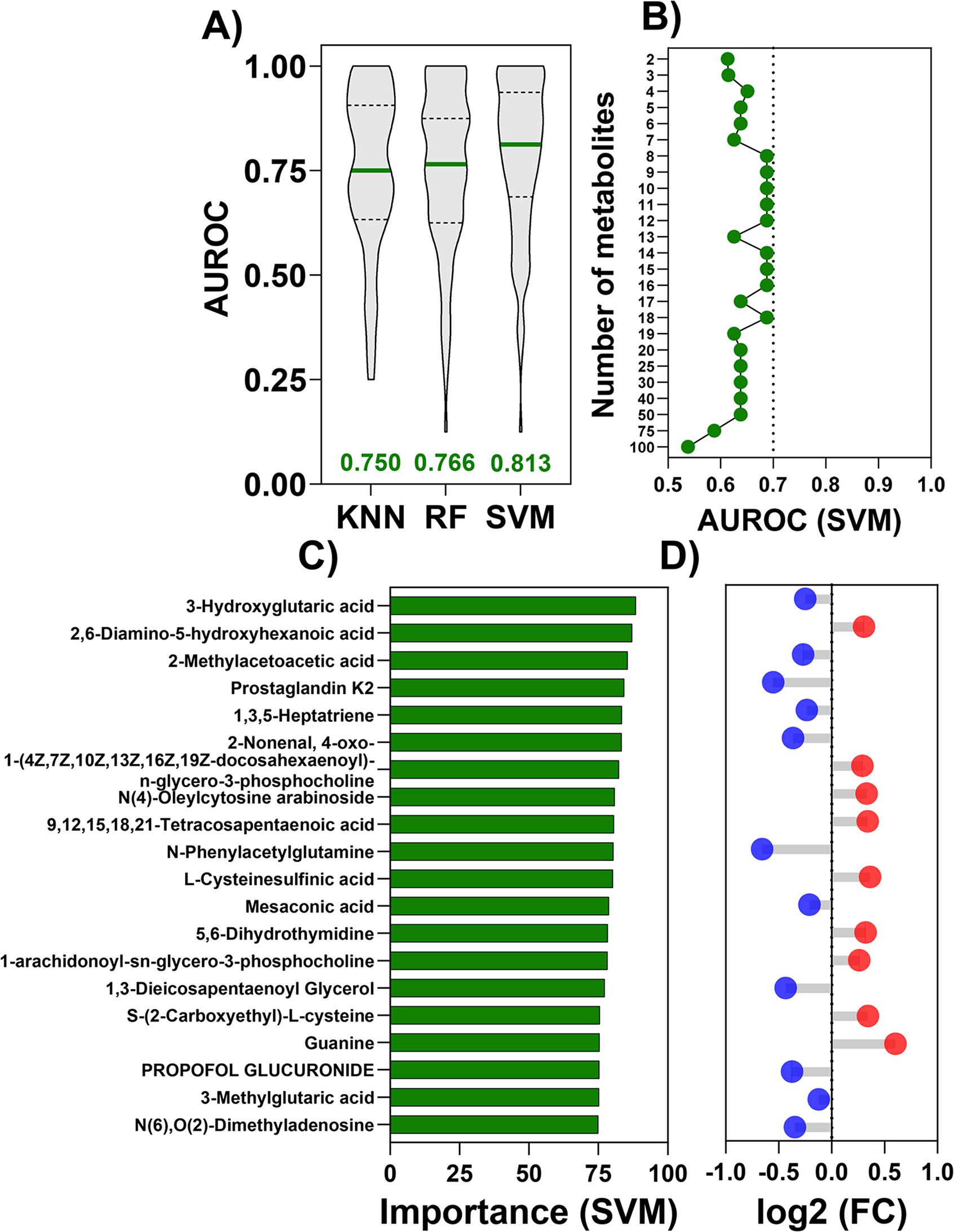
Predicting metabolic markers in MIBC over NMIBC using parsimonious machine learning models. **A**) Three machine learning classification methods: k-nearest neighbor (KNN), Random Forest (RF), and Support Vector Machines (SVM) linear were used to predict MIBC (n = 41) over NMIBC (n = 10); One hundred cross-validation iterations were performed, using 80% of the data as training and 20% as testing. The median area under the receiver operating characteristic curve (AUROC) of each model is listed for each method. **B**) Minimal feature classifiers were identified by selecting metabolite features informative in ≥70% of 100 iterations. SVM classification was performed with an increasing number of features to predict MIBC over NMIBC. Each green dot represents the median AUROC for a given number of metabolites. **C**) The top 20 informative metabolites are plotted and sorted in decreasing order by important features in MIBC over NMIBC. **D**) Lollipop plot shows the fold changes of the top 20 metabolites in MIBC over NMIBC, and data derived from Figure 3B. Detailed statistical analyses, including significance values for these metabolites, are presented in Supplementary Data 2

To complement the analysis of differentially expressed metabolites analysis, we explored the potential of machine learning applications to our untargeted metabolomics BLCA dataset. A challenging problem in the field is determining metabolic markers of MIBC vs NMIBC. A total of 2590 metabolites were used as input features, and the samples were categorized into MIBC (*n* = 41) and NMIBC (*n* = 10). We applied three machine learning classification models; all models demonstrated robust performance, achieving AUROC values of 0.750 for KNN, 0.766 for Random Forest (RF) and 0.813 for Support Vector Machine (SVM) in distinguishing MIBC from NMIBC (Fig. 4A). Based on its superior performance, the SVM model was selected to determine metabolic markers. Informative features were identified in at least 70% of the cross-validation iterations, and feature importance was computed using Interpretable Machine Learning (IML); as few as 8 metabolites sufficed to classify effectively MIBC vs NMIBC (Fig. 4B). The top 20 informative metabolites, ranked by feature importance, are shown in Fig. 4C, and their corresponding fold changes are displayed in Fig. 4D. Notably, 3-hydroxyglutaric acid, 3-methylglutaric acid, mesaconic acid, and 2-methylacetoacetic acid are decreased, and guanine is increased in patients with MIBC (Figs. 4C and 4D). Next, we investigated BLCA over adjacent normal/benign tissue samples; whereas this analysis is less challenging than the MIBC vs NMIBC, we wanted to determine the potential for machine learning to determine and rank informative metabolic markers for BLCA. A total of 2590 metabolites were used as input features, with samples classified as adjacent normal/benign (*n* = 29) or BLCA (*n* = 65). Comparing BLCA to adjacent normal/benign samples, all three models used demonstrated expected robust performance, each achieving an AUROC above 0.90-specifically, 0.938 for KNN, 0.938 for RF, and 0.923 for SVM (Supplementary Figure 7A). Based on its superior performance, the KNN model was selected to determine metabolic markers for BLCA. Informative features identified in at least 70% of the cross-validation iterations were ranked based on feature importance as few as 20 metabolites sufficed to effectively classify BLCA over adjacent normal/benign tissues (Supplementary Figure 7B). The top 20 metabolites, ranked by feature importance, are shown in Supplementary Figure 7C, along with their corresponding fold changes in Supplementary Figure 7D. Adipoylglycine, S-Adenosylmethionine, 2’-O-Methylguanosine, Glutaminylproline, trans-2-Hexacosenoic acid, N-propyl-L-arginine, N-Acetylmuramate, O-Succinyl-L-homoserine, 1-Methylguanosine, and 1,1-Dimethylurea were increased while 4-Thiouridine decreased in BLCA patients compared to adjacent normal/benign (Supplementary Figure 7C and 7D).

## Discussion

Metabolites are central regulators of cellular functions and play critical roles in the initiation and progression of a wide range of diseases, including cancers [46, 47]. The emergence of metabolomics technologies has enabled systematic capture of dynamic metabolic changes under pathological conditions, providing transformative insights into disease mechanisms. Over the past years, the metabolomics field has advanced rapidly, driven by innovations in analytical platforms, acquisition strategies and computational analysis methods [12, 14–18].

Accurately characterizing cellular metabolism is particularly crucial in cancer metabolism, where metabolic reprogramming is now recognized as a hallmark of tumor progression and therapeutic resistance [48–50]. Several analytical platforms are available to interrogate cancer metabolism including Seahorse [12], Nuclear Magnetic Resonance (NMR) [51], and MS-based approaches [12, 14, 16, 17]. While each of the available methods has its merits, they also have distinct limitations. NMR is non-destructive but suffers from relatively low sensitivity and limited dynamic range [52]. MS-based platforms utilizing Electro Spray Ionization (ESI), Matrix-Assisted Laser Desorption/Ionization (MALDI), Desorption Electrospray Ionization (DESI) sources have been widely applied to metabolomics [53–56]. Gas Chromatography (GC)-MS is known for strong separation capacity for volatile compounds, but it often require labor intensive derivatization [57, 58].

In contrast, liquid chromatography coupled with a high-resolution Orbitrap-based instrument-offers high sensitivity, a broad dynamic range, and the ability to detect thousands of metabolites in a single analysis. In this study, we developed and applied a high-throughput untargeted metabolomics platform using high-resolution Orbitrap IQ-X mass spectrometry coupled with liquid chromatography to profile the metabolome in BLCA. By integrating both RP and HILIC in both positive and negative ionization modes, our platform captures the full spectrum of polar and non-polar metabolites, enabling comprehensive metabolic coverage. The Tribrid Orbitrap system used in our study integrates a quadrupole, linear ion trap, and Orbitrap analyzer, enabling ultra-high resolution, sub-ppm mass accuracy, and rapid scan speeds [59]. Advanced acquisition modes such as Data Dependent Acquision (DDA) enhance confidence of metabolites identification, reduce FDR, and enable precise measurement of metabolites.

Our previous studies have employed both targeted and untargeted LC-MS/TOF strategies to investigate the metabolic landscape of BLCA [11]. This effort includes analysis of tumor tissue and serum samples using LC-MS. This study reveals key dysregulated pathways including xenobiotic metabolism [11], amino acid metabolism [60], and fatty acid oxidation across BLCA stages as well as alterations in oxidative phosphorylation, amino acid metabolism and xenobiotic metabolism in BLCA [12, 16, 17, 61, 62]. Collectively, these findings highlight critical metabolic vulnerabilities that may be exploited therapeutically. A subset of the metabolites, either from in-house retention time (RT)-based spectral library or from the NIST spectral database, may represent structurally distinct isoforms. However, annotation of the metabolites, derived from mzCloud or ChemSpider, were mapped to corresponding genes, and subsequent pathway analyses were performed as part of the current study.

Despite these advances, the full scope of metabolic rewiring in BLCA remains incompletely characterized, owing to biological heterogeneity and technical limitations of existing platforms. The analytical depth of our LC–Tribrid MS platform overcomes many of these challenges, enabling comprehensive metabolic profiling of BLCA tumors relative to adjacent normal/benign tissues. In addition, our platform was able to identify distinct metabolic signatures in aggressive MIBC patients. This approach not only validates previous findings, including the well-documented elevation of xenobiotic metabolism in BLCA, but also provides a deeper understanding of the broader metabolic framework underlying the disease.

In parallel, the complexity and volume of data generated by the LC-MS platform demand advanced analytical approaches. Machine Learning (ML) has emerged as a powerful tool to extract meaningful patterns, enable disease prediction, and facilitate patient stratification [31, 63, 64]. ML with metabolomics has been applied to diseases such as cardiovascular [65, 66], gastric cancer [63], lung adenocarcinoma [67], and other diseases [68–70]. In the context of BLCA, we leverage this synergy to identify novel metabolic markers and gain mechanistic insights into tumor biology.

In this study, we employed three ML models namely k-nearest neighbors (KNN), random forest (RF), and support vector machine (SVM). Among the various models, KNN slightly outperformed the others in adjacent normal/benign vs BLCA. Using a KNN machine learning approach, we identified several metabolic markers. Among the key metabolites altered in BLCA patients were adipoylglycine, 2′-O-methylguanosine, S-adenosylmethionine (SAM), and 4-thiouridine. The accumulation of methylated nucleosides such as 2’-O-methylguanosine and the elevated presence of S-adenosylmethionine suggest increased methylation activity, which is commonly associated with epigenetic dysregulation in tumor cells. Additionally, the increased in amino acid derivatives like adipoylglycine may reflect perturbations in nitrogen metabolism, further supporting the notion of reprogrammed metabolic pathways that contribute to cancer progression [28, 71–74]. SAM, a key molecules in cellular metabolism, regulates crucial DNA and protein methylation for gene expression and epigenetics modulation [75–77]. In BLCA, altered SAM metabolism contributes to unusual methylation and activation [78, 79]. These disruptions associate SAM to metabolic and epigenetic reprogramming that promotes tumor progression. Additionally, 2′-O-Methylguanosine is an endogenous RNA methylated nucleoside that is altered in BLCA in our study, which may reflect RNA methylation dysregulation including cell proliferation [80, 81].

Of note, among the various models tested, SVM exhibited slightly superior performance in distinguishing NMIBC from MIBC. Using SVM model, we also identified several metabolic markers metabolites associated with MIBC. An example, 3-Hydroxyglutaric acid, 3-Methylglutaric acid and 2-Methylacetoacetic acid were significantly decreased, while guanine was increased. The reduction of the organic acids may indicate distresses in branched-chain amino acid catabolism and related pathways, which are often linked to energy homeostasis and cellular stress responses [82]. Those changes could reflect a shift toward alternative metabolic routes that support tumor growth and survival. To the best of our knowledge, the suppression of 3-hydroxyglutaric acid, 3-methylglutaric acid, and 2-methylacetoacetic acid has not been explored in BLCA. We hypothesize that this reduction may be associated with cancer-related metabolic reprogramming, whereby tumor cells divert carbon flux away from oxidative catabolism toward anabolic biosynthesis and redox homeostasis to sustain proliferation and survival [83, 84]. Additionally, the pronounced increase in guanine intend to enhanced purine metabolism, consistent with the high nucleotide demand of rapidly proliferating cancer cells [85–87]. Dysregulated purine biosynthesis has been implicated in tumor progression and may contribute to chemoresistance, highlighting its potential as a therapeutic target [85, 88, 89]. In contrast, the elevated guanine levels are consistent with accelerated purine salvage and heightened nucleotide turnover, metabolic programs known to sustain rapid DNA replication, support RNA biogenesis, and enable continuous proliferation in high-grade malignancies [86, 90]. Enhanced purine metabolism also contributes to redox buffering and epigenetic regulation through SAM and folate-linked one-carbon pathways, aligning with known oncogenic metabolic dependencies in BLCA [85, 88, 91]. Our study has a few limitations, the sample size in our study was relatively small which requires validation with a larger number of samples. Additionally, our platform also detected metabolites with high mass accuracy (Category C) which may require further validation in future studies.

Taken together, our LC-MS-based untargeted metabolomics platform offers exceptional analytical depth, resolution, and versatility for investigating cancer metabolism. When coupled with advanced computational analyses, such as machine learning approaches, it provides a powerful framework for discovering metabolic markers in BLCA. This integrative approach could greatly enhance our understanding of tumor metabolism and support early detection, prognosis, and future personalized therapy in BLCA.

## Conclusion

This study presents the successful development and validation of a robust untargeted metabolomics platform leveraging the Thermo IQ-X Orbitrap Tribrid Mass Spectrometer coupled with the UHPLC system. We have applied these advanced platforms to BLCA tissue samples, this platform enabled high-resolution metabolic profiling and revealed distinct metabolic signatures between tumor and adjacent normal/benign tissues, as well as between patients with muscle-invasive bladder cancer (MIBC) and non-muscle-invasive bladder cancer (NMIBC). Our analysis identified novel dysregulated pathways-including xenobiotic metabolism, glutathione metabolism, amino acid metabolism, lipid metabolism, and glycolysis-highlighting potential metabolic vulnerabilities and offered critical insights into BLCA biology and therapeutic targeting. Furthermore, utilizing machine learning approaches holds promise for predicting key metabolic markers for both BLCA diagnosis and their stratification.

## Electronic supplementary material

Below is the link to the electronic supplementary material.

Supplementary Figures (1-7)

Supplementary Data (1-8)

Supplementary Tables (1-9)

## Data Availability

Raw and processed metabolomics data from samples are available at NIH Metabolomics workbench (Study ID: ST003990). All the raw data values used for the study are in supplementary data (1–8).

## Abbreviations

BLCA: Bladder cancer
ML: Machine learning
LC: Liquid chromatography
MS: Mass spectrometry
HILIC: Hydrophilic interaction liquid chromatography
RP: Reverse phase
DDA: Data dependent acquisition
HCD: High energy collisional dissociation
MIBC: Muscle-invasive bladder cancer
NMIBC: Non-muscle-invasive bladder cancer
QC: Quality control
KNN: k-nearest neighbor
RF: Random forest
SVM: Support vector machine
CD: Compound discoverer
KEGG: Kyoto encyclopedia of genes and genomes
GO: Gene ontology
