# Supplementary material for: Leveraging untargeted metabolomics in combination with machine learning to uncover novel insights into bladder cancer
Source: Cancer Metab. 2026 Apr 1;14:8. doi: 10.1186/s40170-026-00427-4 (PMC13040883; doi:10.1186/s40170-026-00427-4)
Supplement: Supplementary file 1 — Supplementary Figures (1-7) [file 40170_2026_427_MOESM1_ESM.pptx]

## Slide 1
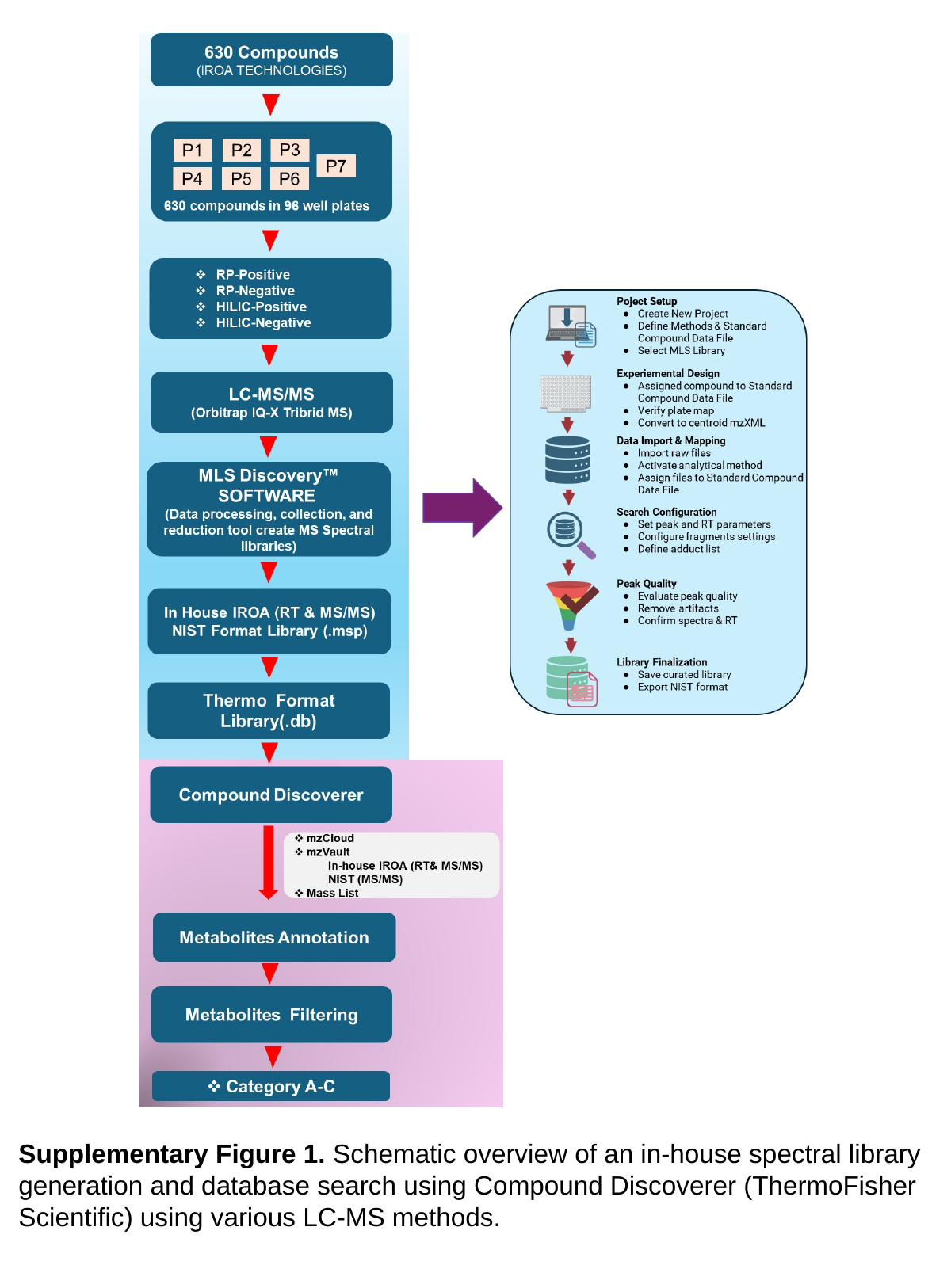

Supplementary Figure 1. Schematic overview of an in-house spectral library generation and database search using Compound Discoverer (ThermoFisher Scientific) using various LC-MS methods.

## Slide 2
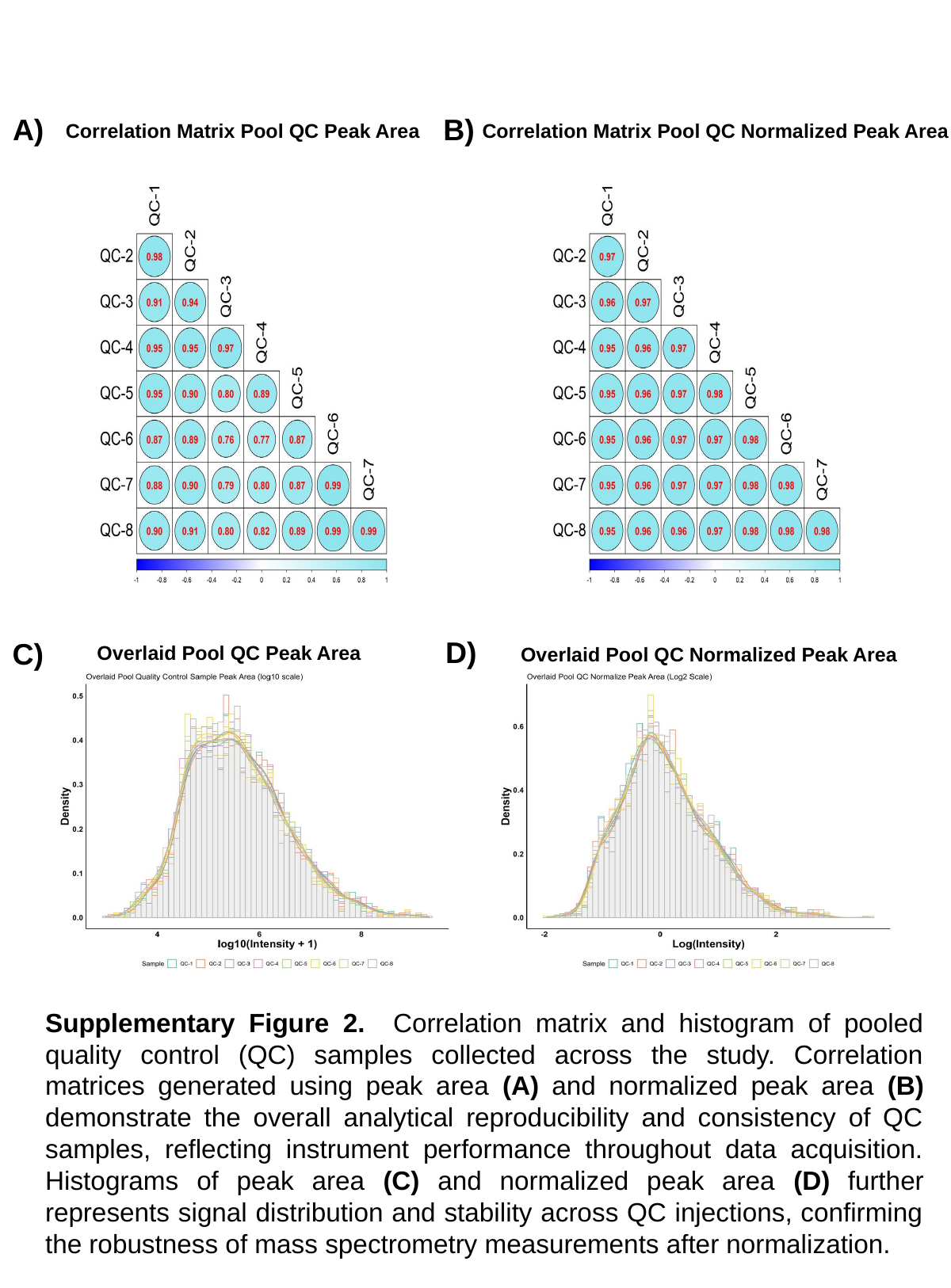

A)
B)
Correlation Matrix Pool QC Peak Area
Correlation Matrix Pool QC Normalized Peak Area
D)
C)
Overlaid Pool QC Peak Area
Overlaid Pool QC Normalized Peak Area
Supplementary Figure 2. Correlation matrix and histogram of pooled quality control (QC) samples collected across the study. Correlation matrices generated using peak area (A) and normalized peak area (B) demonstrate the overall analytical reproducibility and consistency of QC samples, reflecting instrument performance throughout data acquisition. Histograms of peak area (C) and normalized peak area (D) further represents signal distribution and stability across QC injections, confirming the robustness of mass spectrometry measurements after normalization.

## Slide 3
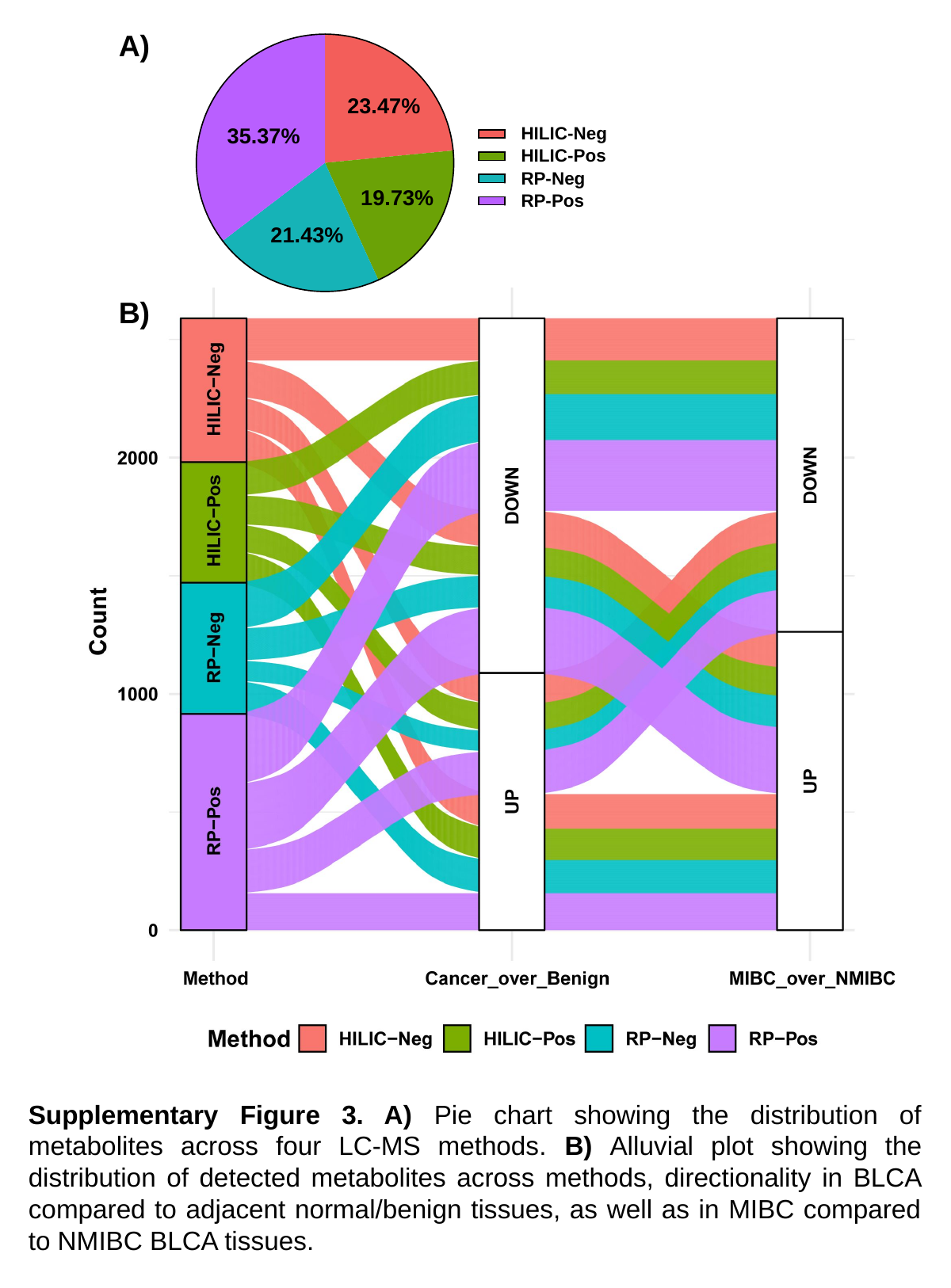

A)
B)
Supplementary Figure 3. A) Pie chart showing the distribution of metabolites across four LC-MS methods. B) Alluvial plot showing the distribution of detected metabolites across methods, directionality in BLCA compared to adjacent normal/benign tissues, as well as in MIBC compared to NMIBC BLCA tissues.

## Slide 4
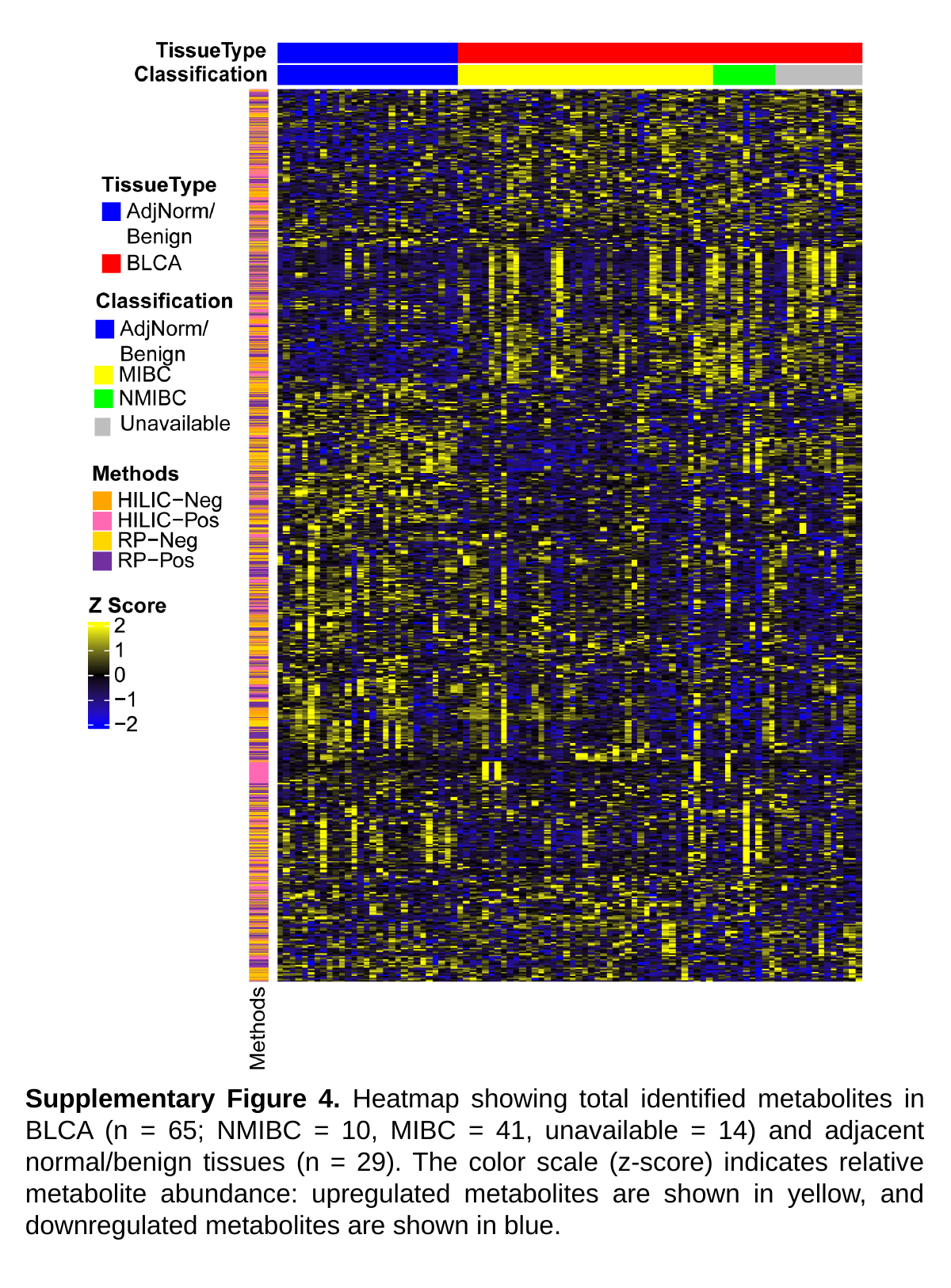

Supplementary Figure 4. Heatmap showing total identified metabolites in BLCA (n = 65; NMIBC = 10, MIBC = 41, unavailable = 14) and adjacent normal/benign tissues (n = 29). The color scale (z-score) indicates relative metabolite abundance: upregulated metabolites are shown in yellow, and downregulated metabolites are shown in blue.

## Slide 5
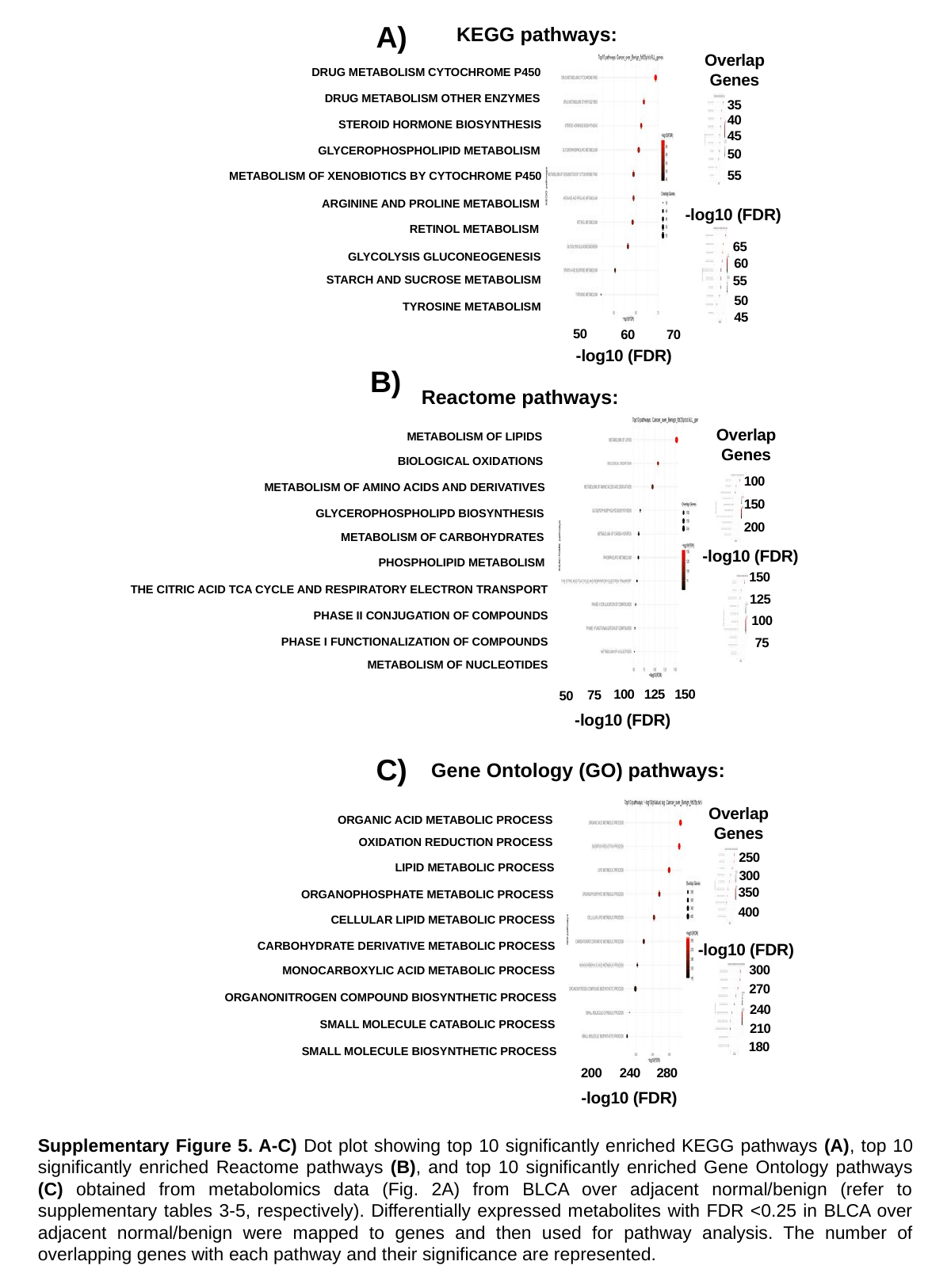

A)
KEGG pathways:
Overlap Genes
DRUG METABOLISM CYTOCHROME P450
DRUG METABOLISM OTHER ENZYMES
35
40
45
50
55
STEROID HORMONE BIOSYNTHESIS
GLYCEROPHOSPHOLIPID METABOLISM
METABOLISM OF XENOBIOTICS BY CYTOCHROME P450
ARGININE AND PROLINE METABOLISM
-log10 (FDR)
RETINOL METABOLISM
65
GLYCOLYSIS GLUCONEOGENESIS
60
STARCH AND SUCROSE METABOLISM
55
50
TYROSINE METABOLISM
45
50
70
60
-log10 (FDR)
B)
Reactome pathways:
Overlap Genes
METABOLISM OF LIPIDS
BIOLOGICAL OXIDATIONS
100
METABOLISM OF AMINO ACIDS AND DERIVATIVES
150
GLYCEROPHOSPHOLIPD BIOSYNTHESIS
200
METABOLISM OF CARBOHYDRATES
-log10 (FDR)
PHOSPHOLIPID METABOLISM
150
THE CITRIC ACID TCA CYCLE AND RESPIRATORY ELECTRON TRANSPORT
125
PHASE II CONJUGATION OF COMPOUNDS
100
PHASE I FUNCTIONALIZATION OF COMPOUNDS
75
METABOLISM OF NUCLEOTIDES
150
125
100
75
50
-log10 (FDR)
C)
Gene Ontology (GO) pathways:
Overlap Genes
ORGANIC ACID METABOLIC PROCESS
OXIDATION REDUCTION PROCESS
250
LIPID METABOLIC PROCESS
300
ORGANOPHOSPHATE METABOLIC PROCESS
350
400
CELLULAR LIPID METABOLIC PROCESS
CARBOHYDRATE DERIVATIVE METABOLIC PROCESS
-log10 (FDR)
MONOCARBOXYLIC ACID METABOLIC PROCESS
300
270
ORGANONITROGEN COMPOUND BIOSYNTHETIC PROCESS
240
SMALL MOLECULE CATABOLIC PROCESS
210
180
SMALL MOLECULE BIOSYNTHETIC PROCESS
200
240
280
-log10 (FDR)
Supplementary Figure 5. A-C) Dot plot showing top 10 significantly enriched KEGG pathways (A), top 10 significantly enriched Reactome pathways (B), and top 10 significantly enriched Gene Ontology pathways (C) obtained from metabolomics data (Fig. 2A) from BLCA over adjacent normal/benign (refer to supplementary tables 3-5, respectively). Differentially expressed metabolites with FDR <0.25 in BLCA over adjacent normal/benign were mapped to genes and then used for pathway analysis. The number of overlapping genes with each pathway and their significance are represented.

## Slide 6
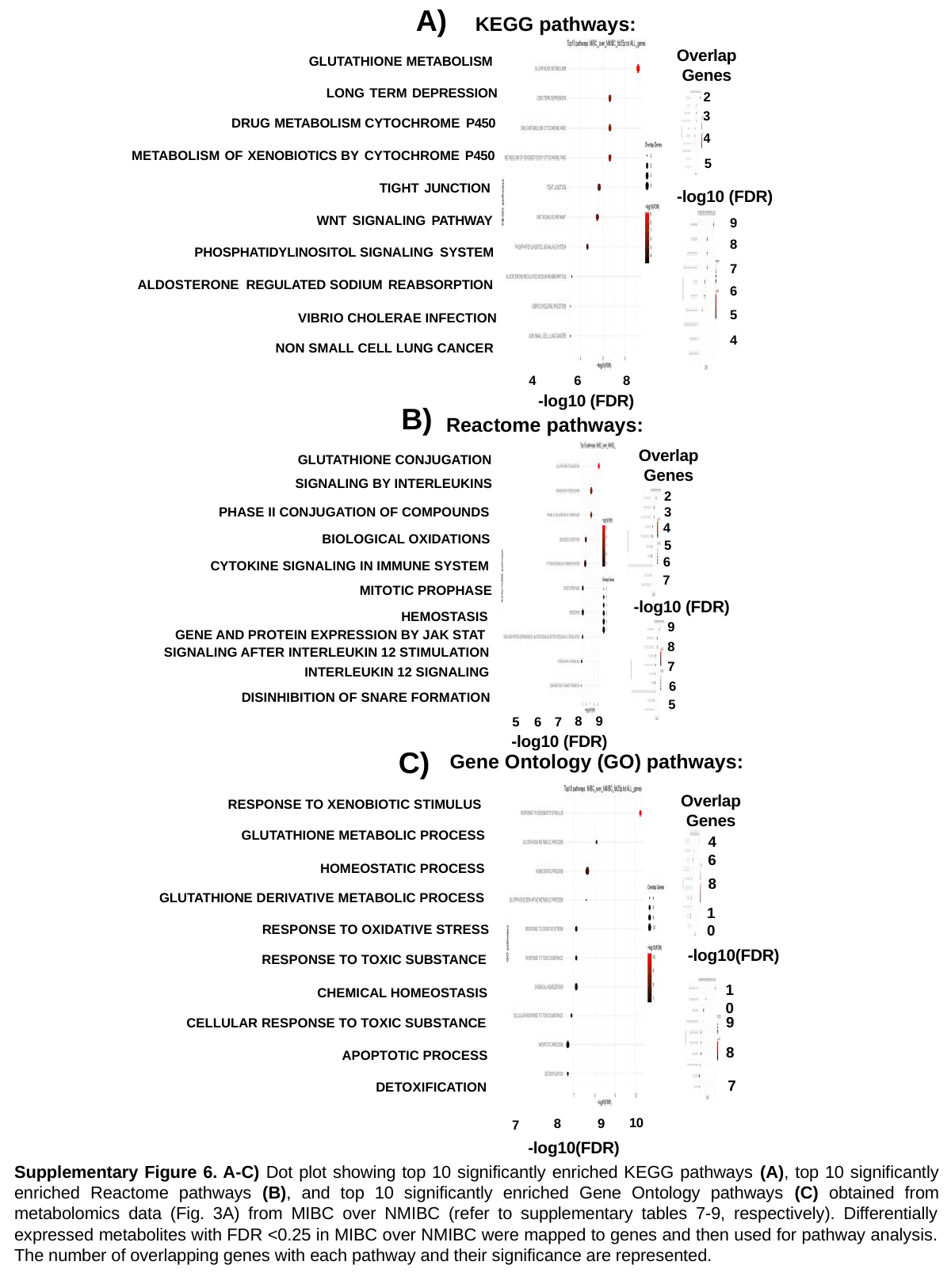

A)
KEGG pathways:
Overlap Genes
GLUTATHIONE METABOLISM
2
LONG TERM DEPRESSION
3
DRUG METABOLISM CYTOCHROME P450
4
METABOLISM OF XENOBIOTICS BY CYTOCHROME P450
5
TIGHT JUNCTION
-log10 (FDR)
9
WNT SIGNALING PATHWAY
8
PHOSPHATIDYLINOSITOL SIGNALING SYSTEM
7
ALDOSTERONE REGULATED SODIUM REABSORPTION
6
5
VIBRIO CHOLERAE INFECTION
4
NON SMALL CELL LUNG CANCER
8
4
6
-log10 (FDR)
B)
Reactome pathways:
Overlap Genes
GLUTATHIONE CONJUGATION
SIGNALING BY INTERLEUKINS
2
3
PHASE II CONJUGATION OF COMPOUNDS
4
BIOLOGICAL OXIDATIONS
5
6
CYTOKINE SIGNALING IN IMMUNE SYSTEM
7
MITOTIC PROPHASE
-log10 (FDR)
HEMOSTASIS
9
GENE AND PROTEIN EXPRESSION BY JAK STAT
SIGNALING AFTER INTERLEUKIN 12 STIMULATION
8
7
INTERLEUKIN 12 SIGNALING
6
DISINHIBITION OF SNARE FORMATION
5
9
8
7
6
5
-log10 (FDR)
C)
Gene Ontology (GO) pathways:
Overlap Genes
RESPONSE TO XENOBIOTIC STIMULUS
GLUTATHIONE METABOLIC PROCESS
4
6
HOMEOSTATIC PROCESS
8
GLUTATHIONE DERIVATIVE METABOLIC PROCESS
10
RESPONSE TO OXIDATIVE STRESS
-log10(FDR)
RESPONSE TO TOXIC SUBSTANCE
10
CHEMICAL HOMEOSTASIS
9
CELLULAR RESPONSE TO TOXIC SUBSTANCE
8
APOPTOTIC PROCESS
7
DETOXIFICATION
10
8
9
7
-log10(FDR)
Supplementary Figure 6. A-C) Dot plot showing top 10 significantly enriched KEGG pathways (A), top 10 significantly enriched Reactome pathways (B), and top 10 significantly enriched Gene Ontology pathways (C) obtained from metabolomics data (Fig. 3A) from MIBC over NMIBC (refer to supplementary tables 7-9, respectively). Differentially expressed metabolites with FDR <0.25 in MIBC over NMIBC were mapped to genes and then used for pathway analysis. The number of overlapping genes with each pathway and their significance are represented.

## Slide 7
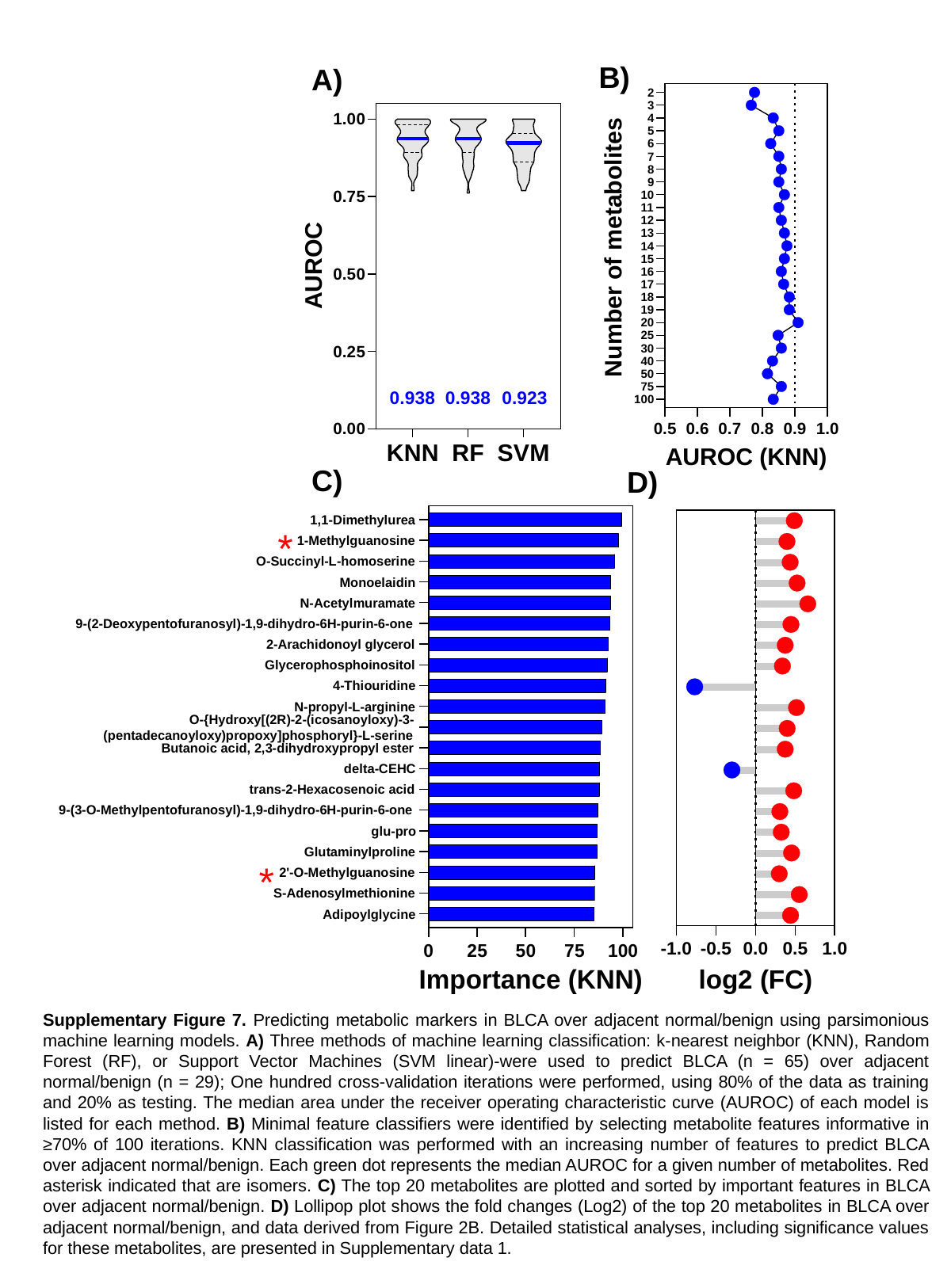

B)
A)
C)
D)
*
*
Supplementary Figure 7. Predicting metabolic markers in BLCA over adjacent normal/benign using parsimonious machine learning models. A) Three methods of machine learning classification: k-nearest neighbor (KNN), Random Forest (RF), or Support Vector Machines (SVM linear)-were used to predict BLCA (n = 65) over adjacent normal/benign (n = 29); One hundred cross-validation iterations were performed, using 80% of the data as training and 20% as testing. The median area under the receiver operating characteristic curve (AUROC) of each model is listed for each method. B) Minimal feature classifiers were identified by selecting metabolite features informative in ≥70% of 100 iterations. KNN classification was performed with an increasing number of features to predict BLCA over adjacent normal/benign. Each green dot represents the median AUROC for a given number of metabolites. Red asterisk indicated that are isomers. C) The top 20 metabolites are plotted and sorted by important features in BLCA over adjacent normal/benign. D) Lollipop plot shows the fold changes (Log2) of the top 20 metabolites in BLCA over adjacent normal/benign, and data derived from Figure 2B. Detailed statistical analyses, including significance values for these metabolites, are presented in Supplementary data 1.
